# Breastfeeding after Anesthesia: A Review for Anesthesia Providers Regarding the Transfer of Medications into Breast Milk

**Published:** 2015

**Authors:** Benjamin Cobb, Renyu Liu, Elizabeth Valentine, Onyi Onuoha

**Affiliations:** Department of Anesthesiology and Critical Care, Perelman School of Medicine at the University of Pennsylvania

## Abstract

Doctors, nurses, and midwives often inform mothers to “pump and dump” their breast milk for 24 hours after receiving anesthesia to avoid passing medications to the infant. This advice, though cautious, is probably outdated. This review highlights the more recent literature regarding common anesthesia medications, their passage into breast milk, and medication effects observed in breastfed infants. We suggest continuing breastfeeding after anesthesia when the mother is awake, alert, and able to hold her infant. We recommend multiple types of medications for pain relief while minimizing sedating medications. Few medications can have sedating effects to the infant, but those medications are specifically outlined. For additional safety, anesthesia providers and patients may screen medications using the National Institute of Health’ LactMed database.

## 1. INTRODUCTION

The positive impact of breastfeeding for both mothers and infants has been well established.^1, 2^ Given the difficulty of conducting randomized clinical trials in breastfeeding mothers and their infants however, there is a paucity of human data regarding the transfer of anesthesia and analgesia medications into breast milk. Available information remains limited to case studies, small sample observations, and animal studies, which may or may not accurately reflect conditions for lactating women.^3, 4^ This lack of knowledge may lead mothers who require medication to abandon nursing on their own accord or based on conservative advice from healthcare practitioners who are concerned about possible adverse effects in the infant.^5^ In addition, physicians, hospitals, and even patients continue to rely on the information given by drug manufacturers concerning the use of drugs during breastfeeding, which is often restrictive or similarly contains contraindications for the lactation period.^4^

The lack of agreement among physicians, the FDA, and hospital policy has created different recommendations and practices regarding, 1) the interval between medication administration and breastfeeding, 2) when to “pump and dump”, and most importantly, 3) which medications are safe. This review highlights the most recent medical literature concerning breastfeeding and the perioperative use of common medications by anesthesia providers during the intraoperative, intrapartum, postoperative and postpartum periods.^6^ The review is focused only on opioid-naïve patients as evidence addressing recommendations in opioid-tolerant lactating mothers remain non-existent. Information presented in this review is written on the premise that data is limited and most medications administered in this patient population have not been rigorously screened by the FDA for safety and efficacy.

## 2. EVIDENCE

The transfer of medications into breast milk depends on pharmacological properties including protein binding, lipid solubility, molecular weight, pKa, and maternal plasma level of the drug.^7^ In general, medications that are highly lipid soluble, less protein-bound, of lower molecular weight, or with a higher pKa are associated with greater penetration into breast milk. The passage of drugs into breast milk most often occurs via passive diffusion proportional to the concentration of drug in maternal plasma. Few drugs are actively transported into breast milk.

### 2.1 The Effect of Intrapartum or Intraoperative Medications

#### A. With Neuraxial Anesthesia

When not contraindicated, neuraxial anesthesia remains the gold standard for both labor analgesia and cesarean delivery.^8^ A combination of local anesthetic and opioid medications are commonly used because their synergistic effect leads to lower required doses for adequate analgesia. The effect of each of these medications on breast-feeding is therefore essential to understand.

##### • Local Anesthesia (LA)

The physicochemical properties of local anesthetics, when used neuraxially, are ideal because these large, polarized molecules do not cross easily into lactating ducts.^3^ Specifically, epidural administrations of lidocaine and bupivacaine have been shown to be safe in lactating mothers. Both were evaluated with their metabolite, pipecolylxylidide (PPX), at 2, 6, and 12 hours in breast milk and maternal serum. The authors concluded that the milk to plasma ratios were consistent with prior data, the transfer of LA and metabolite into breast milk was minimal, and LA can be safely used in breastfeeding mothers. Newborn serum concentrations were unable to be assessed due to transplacental passage of LA, but APGAR scores and 24-hour clinical examinations were normal in the study population.^9^

##### • Opioid

The serum concentration of opioid, which is determined by the dose of spinal or epidural analgesia, is usually minuscule, if at all present. Madej et al found that after a fentanyl bolus of 100 mcg into the epidural space for elective cesarean delivery, the drug in maternal breast milk was undetectable at the time of first request for post-operative analgesics by the mother.^10^ Fentanyl is lipophilic and can be potentially stored in the fatty breast tissue and slowly released into breast milk when used for extended periods of time in the epidural space. Morphine (e.g. hydrophilic opioid) has an oral bioavailability of 30% and its metabolite, morphine-6-glucoride, is pharmacologically active and has a bioavailability of 4%.^6^ This information is relevant because morphine is known to be expressed in breast milk and studies have reported adequate sedation in 50% of infants (AS_50_) with morphine serum concentrations of 125 ng/ml.^11^ Feilberg et al reported a peak morphine concentration of 82 ng/ml in breast milk 30 minutes after a 4mg bolus of morphine given in the epidural space. The authors also report the half-life of breast milk morphine to be 180 minutes.^12^

Given these considerations, both intrathecal or epidural fentanyl and morphine can be safely used for intraoperative analgesia for laboring and cesarean patients intending to breastfeed. However, understanding the pharmacokinetics, limiting the amount of dosed and re-dosed medication, and frequent monitoring of the mother and infant for sedation and respiratory depression is always encouraged. A more conservative approach is that mothers may breastfeed postoperatively as soon as they are adequately alert and oriented to hold the infant because at this point, the breast milk to plasma ratio (M:P) should be minimal.^6^

#### B. With General Anesthesia

It is not uncommon for women to undergo surgical procedures, elective or emergent, in the postpartum period when they are breastfeeding. Questions often arise about the effect of the individual classes of medications used for general anesthesia on breastfeeding.

##### • Induction Agents

1. **Propofol** is an ideal agent for induction of anesthesia in obstetric patients due to its short duration of action, rapid metabolism with no active metabolite, and anti-emetic profile. In a study with 21 patients receiving 2.5 mg/kg induction dose of propofol for elective cesarean section, the authors concluded that breast milk concentrations of propofol were negligible 2 hours following delivery. The low oral bioavailability of propofol and rapid metabolism in infants support the safety of administering breast milk to infants in the immediate postoperative period.^13^
2. **Etomidate** was evaluated in elective cesarean patients’ breast milk 30 minutes and 2 hours after a 0.3 mg/kg induction dose. At 30 minutes post induction, the average breast milk to maternal plasma ratio was 1.2. However, the breast milk concentration decreased 75% at 2 hours and was undetectable at 4 hours. The rapid clearance of etomidate suggests etomidate may be safely used when clinically appropriate for breastfeeding mothers.^14^
3. There are currently no human studies evaluating the transfer of **ketamine** to breast milk.
4. Of the benzodiazepines, **midazolam** is most frequently used by anesthesia providers because of its pharmacokinetic properties including low milk/plasma ratio, low oral bioavailability, ease of titration, hydrophilic nature, quick speed of onset, and short duration of action.^15^ In a study of five breastfeeding mothers, 2 mg of midazolam was administered with general anesthesia and breast milk samples were collected during a 24-hour period and analyzed for midazolam concentration.^16^ A 24-hour breast milk collection contained 0.004% (0.08 ug) of the maternal dose of midazolam. The authors concluded that breast-feeding should not be interrupted because of midazolam usage.

##### • Volatile Agents

There are currently no studies that measure volatile gas levels in human milk following administration of a volatile anesthetic. Nonetheless, evidence exists that inhalational agents are rapidly excreted and have poor bioavailability. Once the partial pressure of gas is removed from the blood, the patient is said to have cleared the volatile agent. This suggests that mothers can resume breastfeeding after inhalational anesthesia without deleterious effects to the infant.^6, 17^

##### • Opioids

Intravenous fentanyl, morphine, and hydromorphone are commonly used opioids for general anesthesia. Fentanyl, approximately one hundred times more potent than morphine, is highly lipophilic and has the ability to be stored in breast tissue. A study of five women receiving 100ug of fentanyl with induction of general anesthesia attempted to demonstrate its safety. The subjects had breast milk samples collected at five evenly dispersed time points over 24 hours. Less that 0.1% of the administered dose was measured in breast milk collection over the time periods or the final combined breast milk. Given the low bioavailability of fentanyl and its short duration of action, the authors recommended resuming breast-feeding in the postoperative period when the mother is alert with reasonable intraoperative fentanyl doses.^16, 18^ There is no literature regarding morphine and hydromorphone as part of a “balanced anesthetic” during cesarean delivery. Their analgesia data and suggestions are provided in the following section on postpartum analgesia. Similarly, there is no literature specifically evaluating remifentanil concentrations in breastmilk. A small study of laboring women who received a patient-controlled remifentanil during delivery demonstrated no difference in neonatal Apgar scores or respiratory outcomes.^19^ Stocki et. al report a very small study of four women who received remifentanil via intravenous infusion during surgery for a target plasma concentration of 1.5 mcg/L at the end of surgery. Infants were then breast fed at 1.5 hours, 2.8 hours, 4.6 hours, and 5 hours after extubation with no untoward respiratory effects noted in the infants.^20^ While small, these studies suggest that remifentanil’s ultra short context sensitive half-life, rapid metabolism by plasma cholinesterase, and high protein binding with low drug free fraction, may make it an ideal narcotic agent for breastfeeding mothers, particularly if postoperative pain is expected to be minimal.^20^

##### • Neuromuscular Blocking Agents

There are no studies to date that evaluate the transfer of neuromuscular blocking agents to breast milk. It is presumed that these agents do not cross the blood-milk duct membranes due to their relatively large size, low lipid solubility, and polarized nature. In addition, the low oral bioavailability of these agents supports their safety in lactating patients.

##### • Reversal of Muscle Relaxant Medications

The standard combination for reversal of neuromuscular paralysis includes an anticholinesterase (neostigmine) with an anticholinergic (glycopyrrolate); less commonly edrophonium and atropine are used, respectively. Neostigmine and glycopyrrolate are quaternary ammonium compounds, which do not penetrate the blood-brain barrier and are not expected to cross into milk ducts.^6, 21^ Of note, neonatal abdominal cramping has been reported in myasthenia gravis patients taking cholinesterase inhibitors while lactating, but serum breast milk cholinesterase levels were undetectable.^21^

#### C. With Regional Anesthesia

Regional anesthesia serves as an alternative or adjuvant for mothers concerned about opioid-based epidural analgesia for labor and future breastfeeding. Although a sub-optimal mode of analgesia for childbirth, paracervical or pudendal blocks and local perineal anesthesia limit the exposure of opioids to the infant. Limitations however, remain its lack of efficacy, technical difficulties in performing them, inability to convert to surgical anesthesia, and a high rate of complications especially with the paracervical block. Given the limited levels that reach systemic circulation and the inability of large molecules to cross into milk ducts, this option of analgesia is very attractive to concerned mothers.^22^

The site of local anesthesia injection is a major determinant of systemic levels of LA. Areas that are highly vascular (e.g. intercostal and epidural space) absorb LA quicker than less vascular areas (e.g. femoral/sciatic and brachial plexus regions). Subcutaneous injection is the least effective at uptake of LA. There are no studies that evaluate the absorption rate of local anesthetics for different regional nerve blocks in breastfeeding mothers. However, one case report demonstrated the use of an intrapleural catheter with bupivacaine (0.25%) for postoperative analgesia following a cholecystectomy in a breastfeeding mother. The infusion rate averaged 10 ml/hr for 5 days. In the immediate post-operative period, the plasma and breast milk concentrations were maximal but found to be undetectable 5 hours later in the infants blood.^23^ Given the physicochemical properties of LA and low oral bioavailability, cessation of breastfeeding is unwarranted when using LA for regional analgesia.

### 2.2 The Effect of Postpartum or Postoperative Medications

“Analgesics” are one of the frequently used classes of medications in the postoperative or postpartum period. Non-opioid analgesics should be selected first for pain management in postpartum women who breastfeed as they are non-sedating for both mother and infant.

Acetaminophen, ibuprofen, and ketorolac are commonly used, especially after cesarean section. Ibuprofen is ideal because the half-life is approximately 1.8 hours. Thus, if mothers medicate themselves at the beginning of breast-feeding, the drug levels are usually negligible if the next feeding is 2-3 hours later.^6, 24^

Meperidine should be avoided due to a theoretical higher risk of neonatal respiratory sedation if taken by breastfeeding mothers in the postpartum period. There are reports of cyanosis, bradycardia, and apnea after its administration.^25^ This is likely due to the presence of meperidine and its metabolite normeperidine with 13 and 65 hour half-lives, respectively. The variability of the half lives also causes a wide range of levels secreted in breast milk.^26^

Codeine, a popular mild analgesic, weakly binds plasma proteins and is highly lipophilic. These pharmacokinetic properties allow for its secretion in breast milk. Codeine must be metabolized in the liver by the CYP450 system into morphine in order to be pharmacologically active. Some mothers may be ultrafast metabolizers, which leads to supratherapeutic morphine levels; infants may also be rapid metabolizers. This can lead to sedating levels of morphine in the breast milk and in extreme cases, respiratory depression and death in the infant.^27^ Therefore, codeine is not recommended for use, especially as other medications are available.

Hydromorphone is approximately seven times more potent than morphine and can be used for acute, extreme pain intramuscularly, intravenously, or orally. There are reports of its transference into breast milk. Following a 2 mg intranasal dose, levels in milk were quite low on a weight-adjusted scale. The dose correlates with about 2.2 ug/day via milk.^28^ The half-life of hydromophone in breast milk was 10.5 hours and hence, discretion should be used given its potency.

Morphine administered orally or via patient controlled analgesia (PCA) must be used cautiously because of the variability of morphine and morphine-6-glucoride in foremilk (colostrum) and hind milk. It has been reported that infants may receive between 0.8–12% of the maternal morphine oral dose and concentrations of up to 1,084 ng/ml of morphine-6-glucoride were found in breast milk of mothers using PCA.^29, 30^ These levels may be dangerous to newborns, infants, and toddlers; and thus close attention must be paid to the infant when mother is receiving this medication. Nevertheless, low-dose morphine is frequently the opioid of choice as passage to breast milk is the less than with other narcotic agents. The bioavailability of morphine is low when taken orally and less is transmitted to the infant after hepatic metabolism in the mother.^31^

An epidural infusion for post-cesarean pain relief minimizes opioid exposure.^32^ A randomized study that compared spinal anesthesia for elective cesarean section with or without the use of postoperative epidural continuous bupivacaine found that the continuous group had lower pain scores and a higher volume of milk fed to their infants.^32^ The authors concluded that mothers that had tolerable pain levels in the postoperative period were more likely to place the infant on their abdomen to breastfeed. The utilization of an epidural infusion for postoperative pain management is also helpful after a major surgical procedure (eg. thoracotomy or open cholecystectomy) where breastfeeding is still desired after surgery.

## 3. CONCLUSION

In summary, codeine and meperidine should be avoided in the lactating mother. Hydromorphone should be utilized with caution. Low-dose morphine can be used safely and is the preferred analgesic in the postoperative or postpartum period.

Given that data is limited and most medications administered in this patient population lack clear evidence on their safety and efficacy profile, a general principal is that a mother can resume breastfeeding once they become awake, stable, and alert after anesthesia has been given.^16^ A return to baseline mentation and strength suggests that sedating medications have redistributed from the plasma and milk compartment to the adipose and muscle and are being slowly released back into the plasma. For additional safety, mothers should closely monitor their infant for signs and symptoms of behavioral changes while consuming medications.

Lastly, patients and providers may exercise additional safety by screening medications using the National Institute of Health’ LactMed database found at http://toxnet.nlm.nih.gov/newtoxnet/lactmed.htm. This peer-reviewed resource provides information regarding drugs transferred into breast milk, safe alternatives to commonly used medications, and other useful facts.
